# Supplementary material for: The microRNA cluster C19MC confers differentiation potential into trophoblast lineages upon human pluripotent stem cells
Source: Nat Commun. 2022 Jun 2;13:3071. doi: 10.1038/s41467-022-30775-w (PMC9163035; doi:10.1038/s41467-022-30775-w)
Supplement: Supplementary file 1 — Supplementary Information [file 41467_2022_30775_MOESM1_ESM.pdf]

<u>**Supplementary Information**</u>

The microRNA cluster C19MC confers differentiation potential into trophoblast lineages upon human pluripotent stem cells

Norio Kobayashi[1], Hiroaki Okae[1,*], Hitoshi Hiura[2], Naoto Kubota[3], Eri H Kobayashi[1], Shun Shibata[1], Akira Oike[1], Takeshi Hori[4], Chie Kikutake[3], Hirotaka Hamada[1], Hirokazu Kaji[4], Mikita Suyama[3], Marie-Line Bortolin-Cavaillé[5], Jérôme Cavaillé[5], Takahiro Arima[1,*]

[1]Department of Informative Genetics, Environment and Genome Research Center, Tohoku University Graduate School of Medicine, Sendai 980-8575, Japan

[2]Department of Bioscience, Faculty of Life Science, Tokyo University of Agriculture, Tokyo 156-8502, Japan

[3]Division of Bioinformatics, Medical Institute of Bioregulation, Kyushu University, Fukuoka 812-8582, Japan

[4]Department of Biomechanics, Institute of Biomaterials and Bioengineering, Tokyo Medical and Dental University, Tokyo 101-0062, Japan

[5]Molecular, Cellular and Developmental biology department (MCD), Centre de Biologie Intégrative (CBI), University of Toulouse, CNRS, UPS, 31062 Toulouse, France

Supplementary Fig. 1

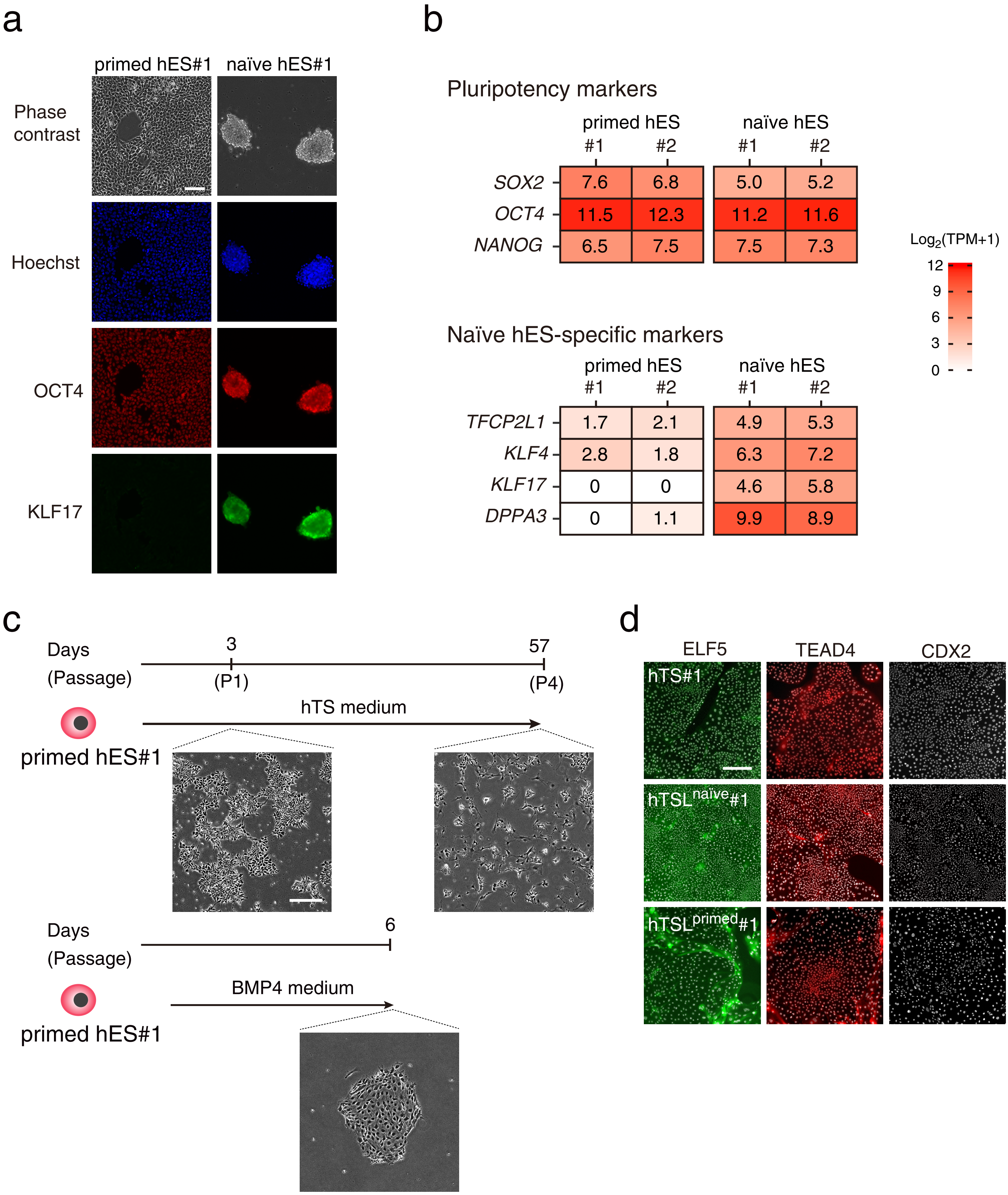

Supplementary Figure 1. Characterization of primed and naïve hES cells and schematic representation of hTSL cell derivation.

(a) Immunostaining of a pluripotency marker (OCT4) and a naïve hES cell-specific marker (KLF17) in primed and naïve hES cells. Nuclei were stained with Hoechst 33258. The scale bar indicates 100 μm. Similar results were obtained with two independent cell lines. (b) Expression levels of pluripotency markers and naïve hES cell-specific markers in primed and naïve hES cells. The data were obtained by RNA-seq. (c) Optimization of the duration of BMP4 treatment. When BMP4 treatment was omitted (upper panel), only cells with mesenchymal morphology proliferated. When primed hES cells were treated with BMP4 for six days (lower panel), cells with epithelial morphology appeared. However, these cells could not survive in hTS medium. The scale bar indicates 300 μm. Similar results were obtained with two independent cell lines. (d) Immunostaining of ELF5, TEAD4, and CDX2 in hTS and hTSL cells. Nuclei were stained with Hoechst 33258 (shown in gray). The scale bar indicates 200 μm. Similar results were obtained with two independent cell lines.

Supplementary Fig. 2

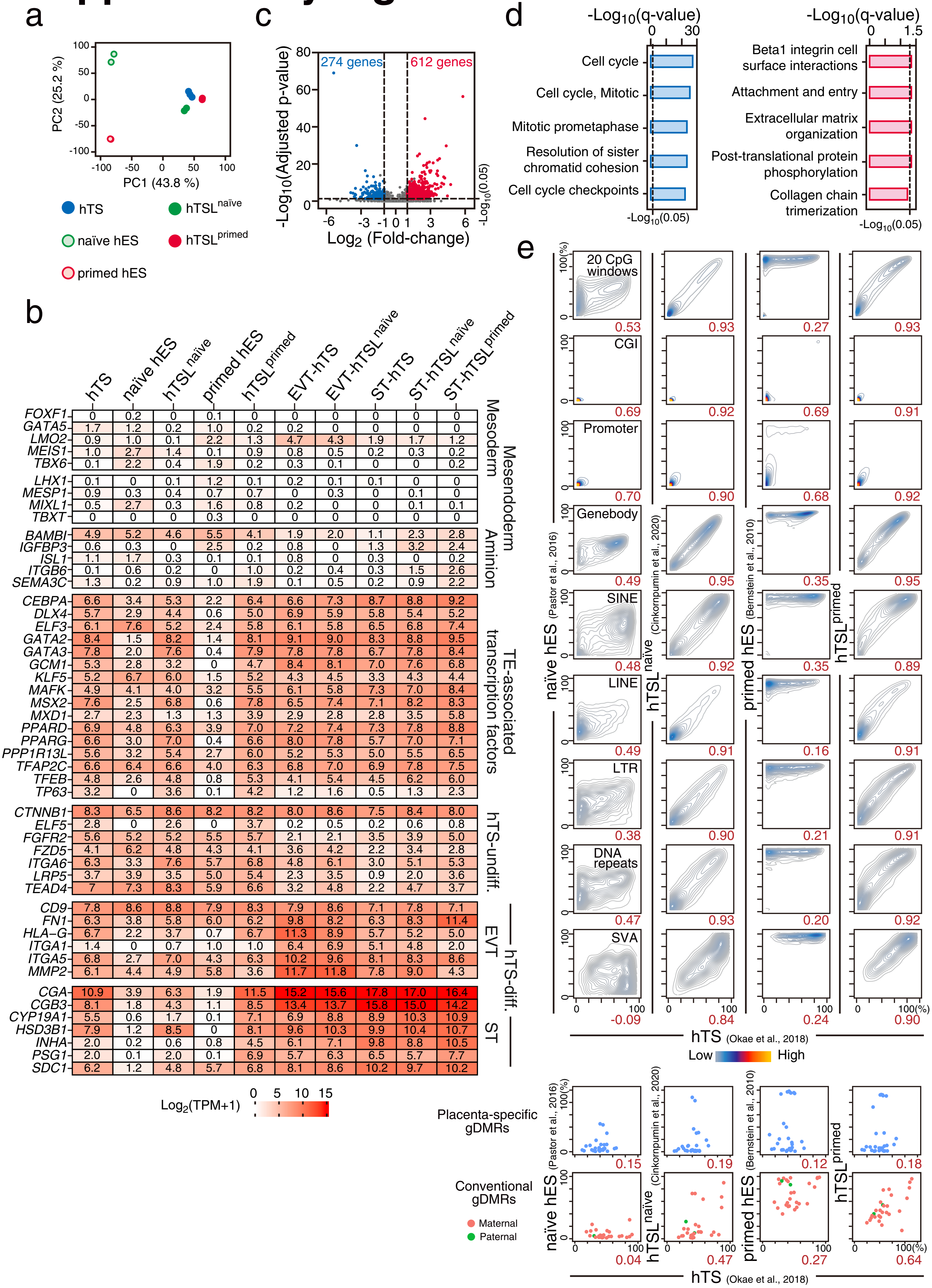

Gene	hTS	naïve hES	hTSL naïve	primed hES	hTSL primed	EVT-hTS	EVT-hTSL naïve	ST-hTS	ST-hTSL naïve	ST-hTSL primed	Group
FOXF1	0	0.2	0	0.1	0	0	0	0	0	0	Mesoderm
GATA5	1.7	1.2	0.2	1.0	0.2	0.2	0	0	0	0	Mesoderm
LMO2	0.9	1.0	0.1	2.2	1.3	4.7	4.3	1.9	1.7	1.2	Mesoderm
MEIS1	1.0	2.7	1.4	0.1	0.9	0.8	0.5	0.2	0.3	0.2	Mesoderm
TBX6	0.1	2.2	0.4	1.9	0.2	0.3	0.1	0	0	0.2	Mesoderm
LHX1	0.1	0	0.1	1.2	0.1	0.2	0.1	0.1	0	0	Mesendoderm
MESP1	0.9	0.3	0.4	0.7	0.7	0	0.3	0	0.1	0	Mesendoderm
MIXL1	0.5	2.7	0.3	1.6	0.8	0.2	0	0	0.1	0.1	Mesendoderm
TBXT	0	0	0	0.3	0	0	0	0	0	0	Mesendoderm
BAMBI	4.9	5.2	4.6	5.5	4.1	1.9	2.0	1.1	2.3	2.8	Aminion
IGFBP3	0.6	0.3	0	2.5	0.2	0.8	0	1.3	3.2	2.4	Aminion
ISL1	1.1	1.7	0.3	0.1	0.1	0.8	0	0.3	0	0.2	Aminion
ITGB6	0.1	0.6	0.2	0	1.0	0.2	0.4	0.3	1.5	2.6	Aminion
SEMA3C	1.3	0.2	0.9	1.0	1.9	0.1	0.5	0.2	0.9	2.2	Aminion
CEBPA	6.6	3.4	5.3	2.2	6.4	6.6	7.3	8.7	8.8	9.2	TE-associated transcription factors
DLX4	5.7	2.9	4.4	0.6	5.0	6.9	5.9	5.8	5.4	5.2	TE-associated transcription factors
ELF3	6.1	7.6	5.2	2.4	5.8	6.1	5.8	6.5	6.8	7.4	TE-associated transcription factors
GATA2	8.4	1.5	8.2	1.4	8.1	9.1	9.0	8.3	8.8	9.5	TE-associated transcription factors
GATA3	7.8	2.0	7.6	0.4	7.9	7.8	7.8	6.7	7.8	8.4	TE-associated transcription factors
GCM1	5.3	2.8	3.2	0	4.7	8.4	8.1	7.0	7.6	6.8	TE-associated transcription factors
KLF5	5.2	6.7	6.0	1.5	5.2	4.3	4.5	3.3	4.3	4.4	TE-associated transcription factors
MAFK	4.9	4.1	4.0	3.2	5.5	6.1	5.8	7.3	7.0	8.4	TE-associated transcription factors
MSX2	7.6	2.5	6.8	0.6	7.8	6.5	7.4	7.1	8.2	8.3	TE-associated transcription factors
MXD1	2.7	2.3	1.3	1.3	3.9	2.9	2.8	2.8	3.5	5.8	TE-associated transcription factors
PPARD	6.9	4.8	6.3	3.9	7.0	7.2	7.4	7.3	7.8	8.8	TE-associated transcription factors
PPARG	6.6	3.0	7.0	0.4	6.6	8.0	7.8	5.7	7.0	7.1	TE-associated transcription factors
PPP1R13L	5.6	3.2	5.4	2.7	6.0	5.2	5.3	5.0	5.5	6.5	TE-associated transcription factors
TFAP2C	6.6	6.4	6.6	4.0	6.3	6.8	7.0	6.9	7.8	7.5	TE-associated transcription factors
TFEB	4.8	2.6	4.8	0.8	5.3	4.1	5.4	4.5	6.2	6.0	TE-associated transcription factors
TP63	3.2	0	3.6	0.1	4.2	1.2	1.6	0.5	1.3	2.3	TE-associated transcription factors
CTNNB1	8.3	6.5	8.6	8.2	8.2	8.0	8.6	7.5	8.4	8.0	hTS-undiff.
ELF5	2.8	0	2.6	0	3.7	0.2	0.5	0.2	0.6	0.8	hTS-undiff.
FGFR2	5.6	5.2	5.2	5.5	5.7	2.1	2.1	3.5	3.9	5.0	hTS-undiff.
FZD5	4.1	6.2	4.8	4.3	4.1	3.6	4.2	2.2	3.4	2.8	hTS-undiff.
ITGA6	6.3	3.3	7.6	5.7	6.8	4.8	6.1	3.0	5.1	5.3	hTS-undiff.
LRP5	3.7	3.9	3.5	5.0	5.4	2.3	3.5	0.9	2.0	3.6	hTS-undiff.
TEAD4	7	7.3	8.3	5.9	6.6	3.2	4.8	2.2	4.7	3.7	hTS-undiff.
CD9	7.8	8.6	8.8	7.9	8.3	7.9	8.6	7.1	7.8	7.1	hTS-diff. / EVT
FN1	6.3	3.8	5.8	6.0	6.2	9.8	8.2	6.3	8.3	11.4	hTS-diff. / EVT
HLA–G	6.7	2.2	3.7	0.7	6.7	11.3	8.9	5.7	5.2	5.0	hTS-diff. / EVT
ITGA1	1.4	0	0.7	1.0	1.0	6.4	6.9	5.1	4.8	2.0	hTS-diff. / EVT
ITGA5	6.8	2.7	7.0	4.3	6.3	10.2	9.6	8.1	8.3	8.6	hTS-diff. / EVT
MMP2	6.1	4.4	4.9	5.8	3.6	11.7	11.8	7.8	9.0	4.3	hTS-diff. / EVT
CGA	10.9	3.9	6.3	1.9	11.5	15.2	15.6	17.8	17.0	16.4	hTS-diff. / ST
CGB3	8.1	1.8	4.3	1.1	8.5	13.4	13.7	15.8	15.0	14.2	hTS-diff. / ST
CYP19A1	5.5	0.6	1.7	0.1	7.1	6.9	8.8	8.9	10.3	10.9	hTS-diff. / ST
HSD3B1	7.9	1.2	8.5	0	8.1	9.6	10.3	9.9	10.4	10.7	hTS-diff. / ST
INHA	2.0	0.2	0.6	0.8	4.5	6.1	7.1	9.8	8.8	10.5	hTS-diff. / ST
PSG1	2.0	0.1	2.0	0.1	6.9	5.7	6.3	6.5	5.7	7.7	hTS-diff. / ST
SDC1	6.2	1.2	4.8	5.7	6.8	8.1	8.6	10.2	9.7	10.2	hTS-diff. / ST

Supplementary Figure 2. Expression levels of lineage markers and DNA methylation levels of various genomic features in hTSL cells.

(a) Principal component analysis (PCA) of hES, hTS, and hTSL cells. Only 8,658 differentially expressed genes (TPM > 4 in at least one cell type, adjusted p-value < 0.05, fold change > 2) were analyzed. (b) Expression levels of lineage markers in hTSL cells and their differentiated derivatives. We performed RNA-seq and analyzed mesoderm and mesendoderm markers[1], amnion markers[2, 3], TE-associated transcription factors[4], and markers for undifferentiated and differentiated hTS cells[5]. (c) Volcano plot comparing $hTSL^{primed}$ cells with hTS and $hTSL^{naïve}$ cells. Gene expression levels in $hTSL^{primed}$ cells were compared to those in hTS and $hTSL^{naïve}$ cells, and expressed as log2 fold change. The down- and up-regulated genes are shown in blue and red, respectively (TPM > 4 in at least one cell type, adjusted p-value < 0.05, fold change > 2). (d) Pathways enriched among the 274 down-regulated and 612 up-regulated genes (blue and red dots in Supplementary Fig. 2c). The top five pathways are shown with q-values. (e) Comparisons of DNA methylation levels of various genomic features between hES, hTS, and hTSL cells. Global DNA methylation patterns were compared using 20 CpG windows and represented as contour plots (top). Contour plots for CGI, promoters, gene bodies, and retrotransposons (SINE, LINE, LTR, DNA repeats, and SVA) are also represented (top). DNA methylation levels of placenta-specific and conventional gDMRs are displayed as scatter plots (bottom). Red numbers indicate Pearson correlation coefficients. Publicly available WGBS data were used for hTS cells[5], naïve hES cells[6], $hTSL^{naïve}$ cells[2], and primed hES cells[7].

Supplementary Fig. 3

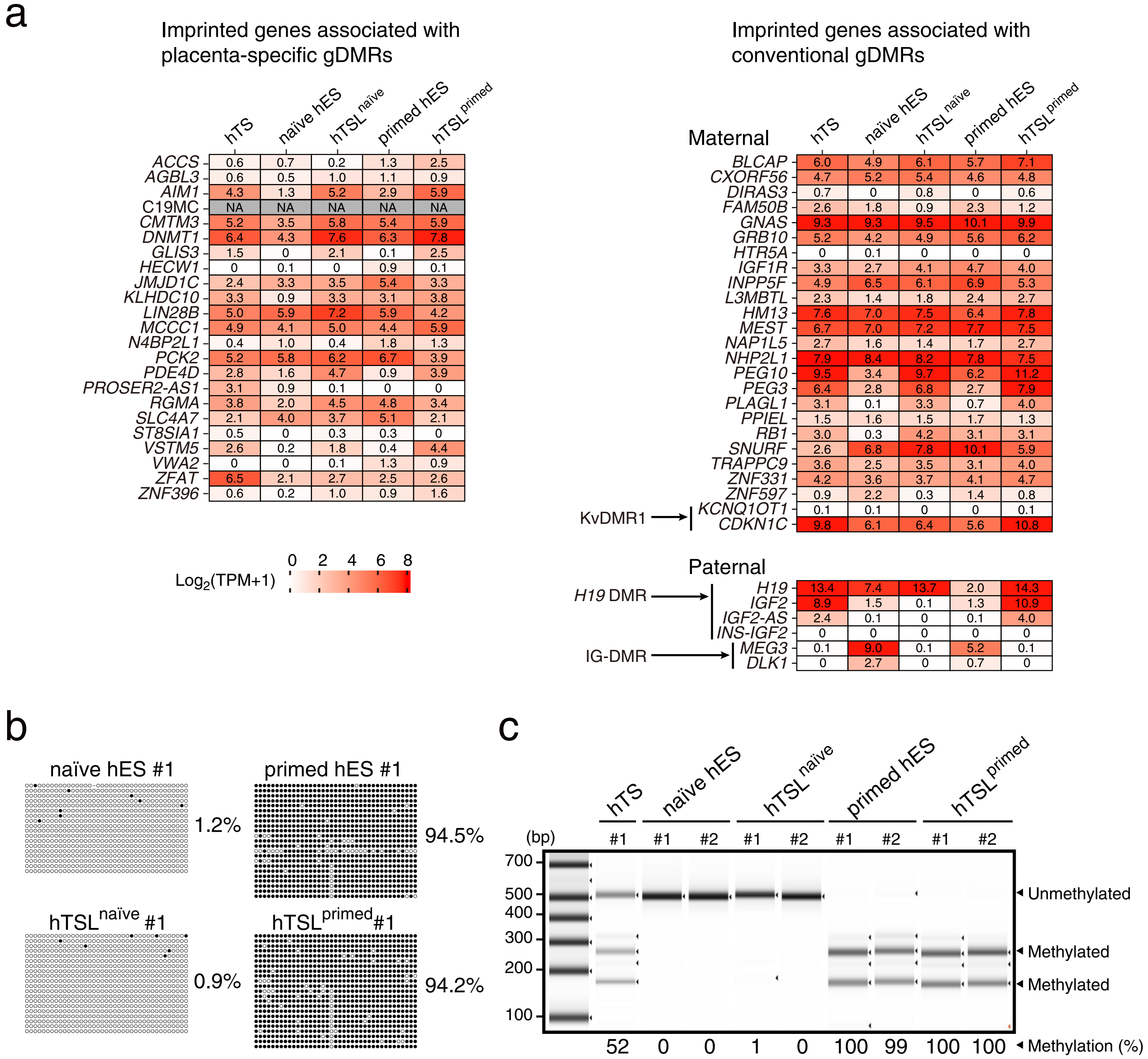

Supplementary Figure 3. Expression levels of imprinted genes in hTSL cells.

(a) Heatmap representation of expression levels of imprinted genes in hES, hTS, and hTSL cells. Note that more than one imprinted gene is shown for the KvDMR1, *H19* DMR, and IG-DMR. (b) DNA methylation analysis of the C19MC DMR by bisulfite sequencing. Black and white circles indicate methylated and unmethylated CpGs, respectively. Methylation levels are shown on the right. (c) DNA methylation analysis of the C19MC DMR by COBRA. The bisulfite-PCR amplicons were digested with HhaI. The C19MC DMR is imprinted in hTS#1 cells[5] and these cells were used as control.

Supplementary Fig. 4

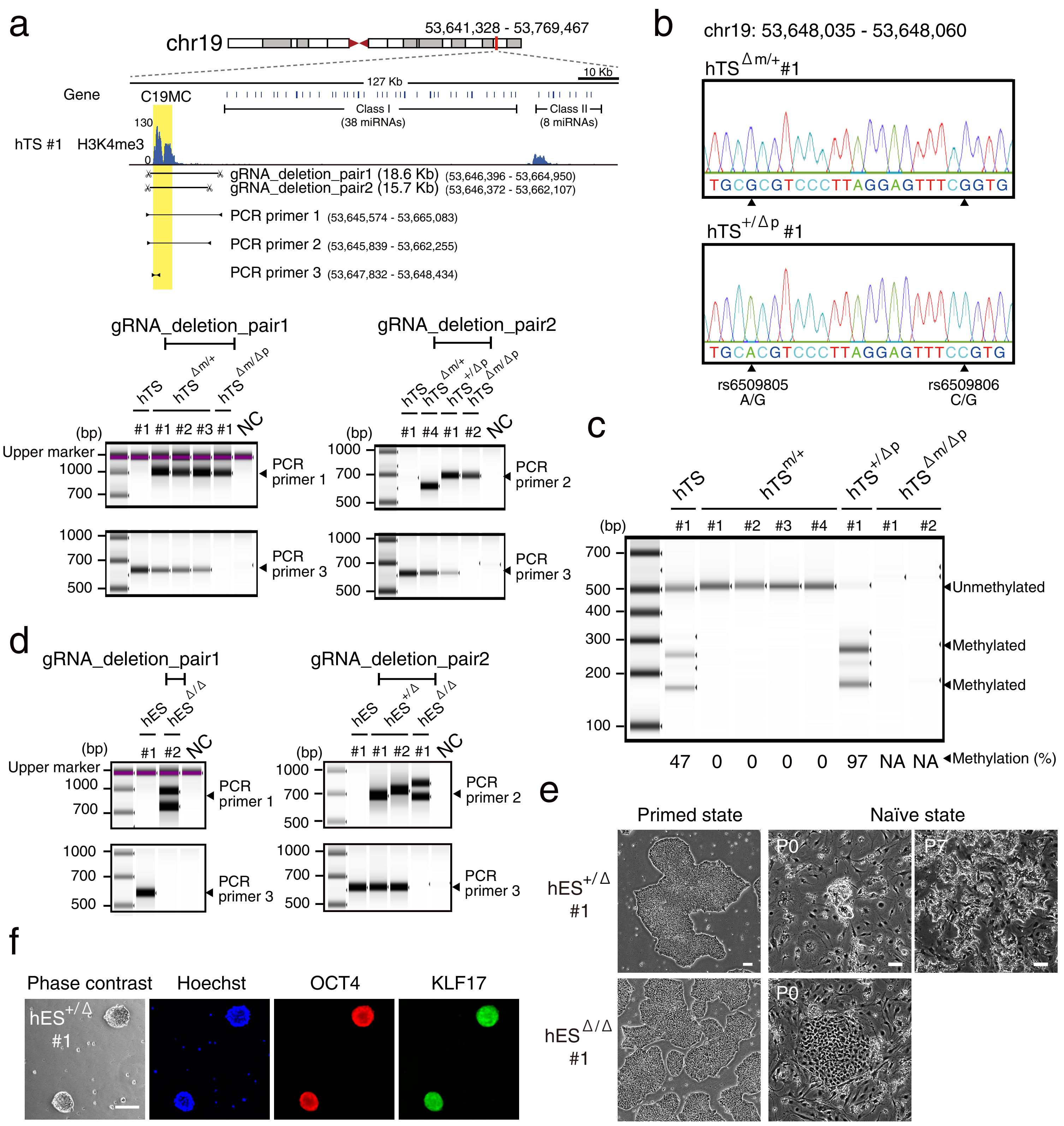

Supplementary Figure 4. Confirmation of the deletion of the C19MC DMR.

(a) Confirmation of the deletion of the C19MC DMR by genomic PCR. The C19MC DMR was deleted in hTS cells using two pairs of gRNAs (gRNA_deletion_pair1 and 2). Deletions were detected by genomic PCR. The position of three PCR primer sets is indicated. Three maternal and one homozygous deletion clone were obtained using gRNA_deletion_pair1. One maternal, one paternal, and one homozygous deletion clone were isolated using gRNA_deletion_pair2. (b) Determination of the deleted alleles using Sanger sequencing in heterozygous deletion clones. Genomic DNA was amplified using the PCR primer set 3. SNPs that were used to distinguish between maternal and paternal alleles are indicated by arrowheads. Four $hTS^{\Delta m/+}$ and one $hTS^{+/\Delta p}$ cell lines were analyzed, and representative results are shown. (c) DNA methylation analysis of the C19MC DMR by COBRA. The bisulfite-PCR amplicons were digested with HhaI. Genetically unmodified hTS cells were used as control. NA: not applicable. (d) Confirmation of the deletion of the C19MC DMR in primed hES cells by genomic PCR. The C19MC DMR was deleted in primed hES cells using two pairs of gRNAs (gRNA_deletion_pair1 and 2). We obtained two heterozygous ($hES^{+/\Delta}$) and two homozygous ($hES^{\Delta/\Delta}$) deletion clones. (e) Derivation of naïve hES cells from primed $hES^{+/\Delta}$ and $hES^{\Delta/\Delta}$ cells using 5i/L/A medium. Naïve hES cells were successfully derived from primed $hES^{+/\Delta}$ cells. However, primed $hES^{\Delta/\Delta}$ cells formed flattened colonies at P0, and these colonies failed to proliferate after passaging. Similar results were obtained with each of the two independent $hES^{+/\Delta}$ and $hES^{\Delta/\Delta}$ clones. The scale bar indicates 100 μm. (f) Immunostaining of a pluripotency marker (OCT4) and a naïve hES cell-specific marker (KLF17) in naïve $hES^{+/\Delta}$ cells. Nuclei were stained with Hoechst 33258. The scale bar indicates 100 μm. Similar results were obtained with two independent $hES^{+/\Delta}$ clones.

Supplementary Fig. 5

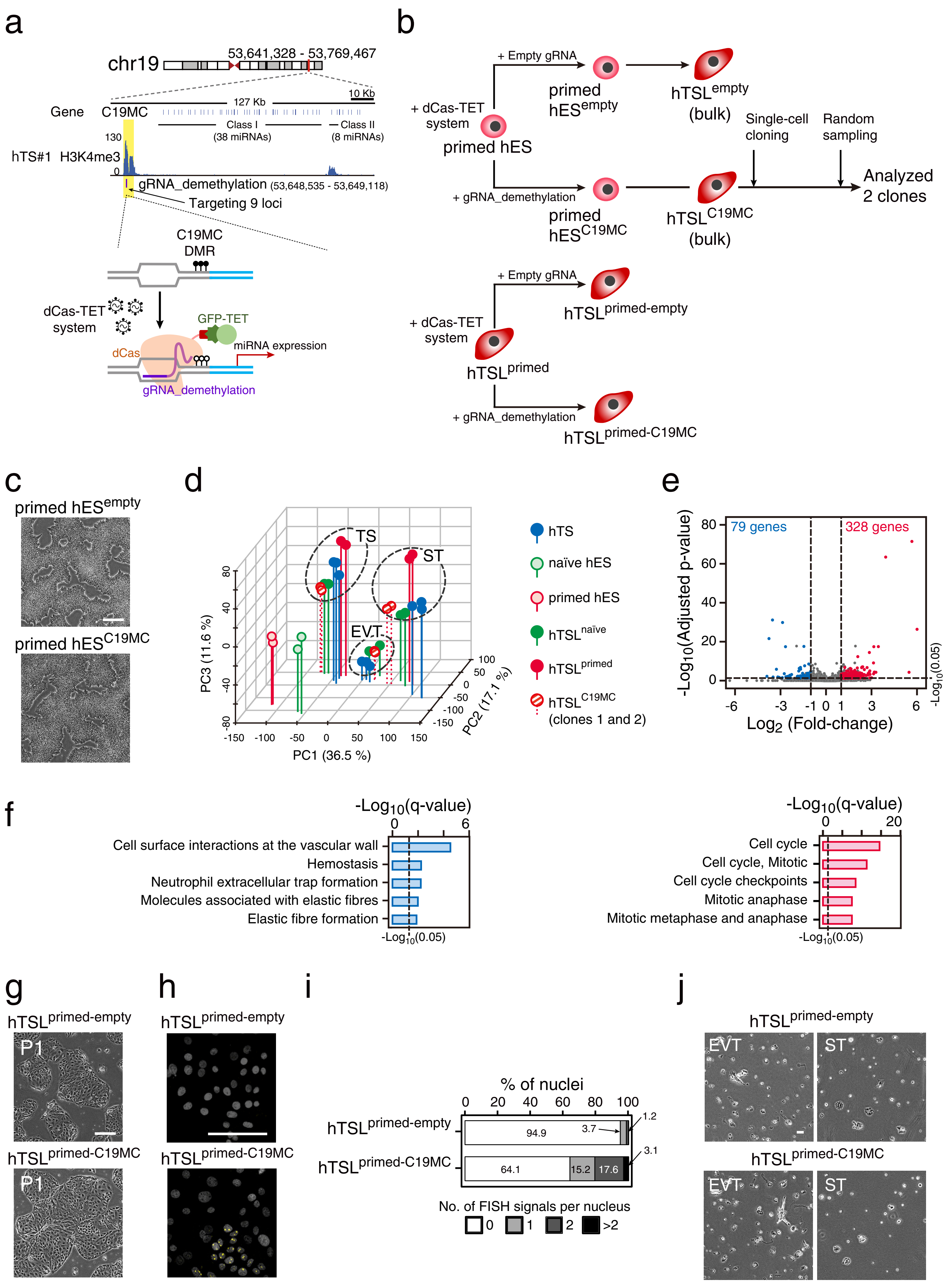

Supplementary Figure 5. Reactivation of C19MC in primed hES and hTSL^{primed} cells.

(a) Schematic representation of C19MC reactivation using the dCas-TET system. The catalytically inactive Cas9 (dCas) is fused to a peptide repeat that can recruit scFv-fused GFP and the catalytic domain of TET1. We designed a gRNA (gRNA_demethylation) targeting the short tandem repeats within the C19MC DMR. The C19MC DMR contains 9 target sites for this gRNA. By combining these components, we demethylated the C19MC DMR. (b) Schematic representation of the derivation of hTSL$^{\text{C19MC}}$ and hTSL$^{\text{primed-C19MC}}$ cells. We delivered the gRNA and the dCas-TET expression vectors into a primed hES cells and derived hTSL$^{\text{C19MC}}$ cells. We further isolated two C19MC-positive clones from these bulk hTSL$^{\text{C19MC}}$ cells by single-cell cloning. As a negative control, we used an empty gRNA vector and derived hTSL^{empty} cells. hTSL$^{\text{primed-C19MC}}$ cells were generated from hTSL^{primed} cells that were originated from genetically unmodified primed hES cells. (c) Phase-contrast images of C19MC-reactivated hES cells. Similar results were obtained with two independent experiments. The scale bar indicates 300 μm. (d) PCA of hTS and hTSL cells. hTSL$^{\text{C19MC}}$ cells had transcriptome profiles similar to hTS and hTSL^{naïve} cells both before and after differentiation. Only 12,853 differentially expressed genes (TPM > 4 in at least one cell type, adjusted p-value < 0.05, fold change > 2) were analyzed. (e) Volcano plot comparing ST-hTSL$^{\text{C19MC}}$ cells with ST-hTS and -hTSL^{naïve} cells. Gene expression levels were compared and expressed as log2 fold change. The down- and up-regulated genes are shown in blue and red dots, respectively (TPM > 4 in at least one cell type, adjusted p-value < 0.05, fold change > 2). (f) Pathways enriched among the genes down- and up-regulated in ST-hTSL$^{\text{C19MC}}$ cells (blue and red dots in Supplementary Fig. 5e). The top five pathways of the down- and up-regulated genes are represented with q-values on the left and right charts, respectively. (g) Phase-contrast images of hTSL$^{\text{primed-empty}}$ and hTSL$^{\text{primed-C19MC}}$ cells. Similar results were obtained with two independent experiments. The scale bar indicates 300 μm. (h) RNA-FISH of C19MC in hTSL$^{\text{primed-empty}}$ and hTSL$^{\text{primed-C19MC}}$ cells. C19MC expression is shown in yellow, and nuclei are in gray. The scale bar indicates 100 μm. (i) Percentage of the number of C19MC FISH signals per nucleus in hTSL$^{\text{primed-empty}}$ and hTSL$^{\text{primed-C19MC}}$ cells. 500-1000 nuclei were examined for each cell type. (j) Phase-contrast images of EVT- and ST-like cells derived from hTSL$^{\text{primed-empty}}$ and hTSL$^{\text{primed-C19MC}}$ cells. Similar results were obtained with two independent experiments. The scale bar indicates 100 μm.

Supplementary Fig. 6

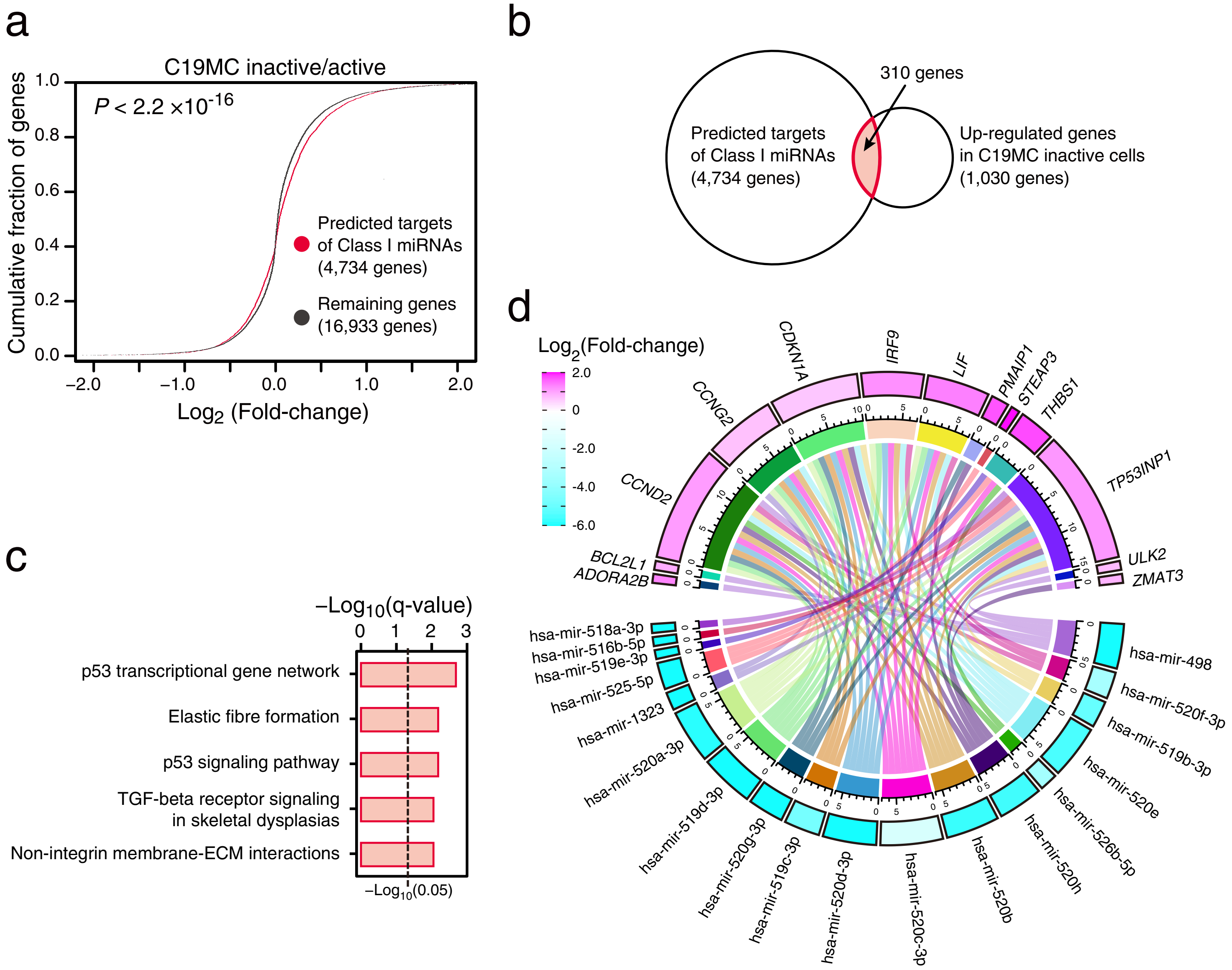

Supplementary Figure 6. Prediction of genes targeted by C19MC miRNAs.

(a) Potential target genes of Class I miRNAs located between the C19MC DMR and the putative second promoter. Target genes were predicted using mirDIP[8] by comparing the gene expression levels in C19MC active (hTS, $hTSL^{naïve}$, $hTS^{\Delta m/+}$, and $hTSL^{C19MC}$) cells with those in C19MC inactive ($hTSL^{primed}$, $hTS^{+/\Delta p}$, $hTS^{\Delta m/\Delta p}$, and $hTSL^{empty}$) cells. The data are expressed as the log2 fold change. Up- and down-regulated genes have positive and negative values, respectively. We considered only genes with Transcripts Per Million (TPM) values greater than zero in at least one cell type. Statistical analysis was performed by two-sided Kolmogorov-Smirnov test. (b) Venn diagram showing the most likely target genes of Class I miRNAs. The gene expression levels in C19MC inactive ($hTSL^{primed}$, $hTS^{+/\Delta p}$, $hTS^{\Delta m/\Delta p}$, and $hTSL^{empty}$) cells were compared with those in C19MC active (hTS, $hTSL^{naïve}$, $hTS^{\Delta m/+}$ and $hTSL^{C19MC}$) cells, and 1,030 up-regulated genes were defined as follows: TPM > 4 in at least one cell type, adjusted p-value < 0.05, and fold change > 1.5. By merging these up-regulated genes with the computationally predicted target genes in (a), we identified 310 genes as the most likely candidates. (c) Overrepresented pathways among the most likely target genes of Class I miRNAs. The top five pathways are represented with q-values. (d) Circos plot of the miRNA-mRNA combinations associated with "p53 transcriptional gene network" and "p53 signaling pathway". The expression levels in C19MC active cells were compared to those in C19MC inactive cells and expressed as the log2 fold change.

Supplementary Table 1. List of the antibodies used in this study.

Description	Company	Catalog number	Dilution rate (Usage)
**Primary antibody**			
Anti-TP63	Cell Signaling	#13109	1:100 (Immunostaining)
Anti-TFAP2C	Santa Cruz Biotechnology	#sc-12762	1:200 (Immunostaining)
Ant-KRT7	Abcam	#ab119697	1:100 (Flow cytometry)
Anti-hCG	DAKO	#IR508	1:10 (Immunostaining)
Anti-KLF17	Sigma	#HPA024629	1:200 (Immunostaining)
Anti-H3K4me3	MBL	#MABI0304	0.8 ng/ul (ChIP)
Anti-ELF5	Sigma	#HPA062706	1:100 (Immunostaining)
Anti-TEAD4	Abcam	#ab58310	1:100 (Immunostaining)
Anti-CDX2	Abcam	#ab157524	1:100 (Immunostaining)
PE-conjugated anti-OCT-4A	Cell Signaling	#56159	1:800 (Immunostaining)
PE-conjugated anti-HLA-G	Abcam	#ab24384	1:50 (Flow cytometry)<br>1:200 (Immunostaining)
PE-conjugated anti-SDC1	Miltenyi Biotec	#130-119-928	1:500 (Immunostaining)
Alexa Fluor 488-conjugated anti-HLA-ABC	Biolegend	#311415	1:50 (Flow cytometry)
**Secondary antibody**			
PE-conjugated mouse IgG1	R&D	#IC006P	
Alexa Fluor 488-conjugated anti-rabbit IgG	Cell Signaling	#4412	
Alexa Fluor 488-conjugated anti-mouse IgG2a	Biolegend	#400233	
Alexa Fluor 555-conjugated anti-mouse IgG	Cell Signaling	#4409	
Alexa Fluor 647-conjugated anti-rabbit IgG	Cell Signaling	#4414	
HRP-conjugated anti-rabbit IgG	Cell Signaling	#7074	

Supplementary References

1. Bernardo, A. S., et al. BRACHYURY and CDX2 mediate BMP-induced differentiation of human and mouse pluripotent stem cells into embryonic and extraembryonic lineages. *Cell Stem Cell* **9**, 144-155 (2011).
2. Cinkornpumin, J. K., et al. Naive Human Embryonic Stem Cells Can Give Rise to Cells with a Trophoblast-like Transcriptome and Methylome. *Stem Cell Rep.* **15**, 198-213 (2020).
3. Guo, G., et al. Human naive epiblast cells possess unrestricted lineage potential. *Cell Stem Cell* **28**, 1040-1056 e1046 (2021).
4. Bai, Q., et al. Dissecting the first transcriptional divergence during human embryonic development. *Stem Cell Rev. Rep.* **8**, 150-162 (2012).
5. Okae, H., et al. Derivation of Human Trophoblast Stem Cells. *Cell Stem Cell* **22**, 50-63 e56 (2018).
6. Pastor, W. A., et al. Naive Human Pluripotent Cells Feature a Methylation Landscape Devoid of Blastocyst or Germline Memory. *Cell Stem Cell* **18**, 323-329 (2016).
7. Bernstein, B. E., et al. The NIH Roadmap Epigenomics Mapping Consortium. *Nat. Biotechnol.* **28**, 1045-1048 (2010).
8. Tokar, T., et al. mirDIP 4.1-integrative database of human microRNA target predictions. *Nucleic Acids Res.* **46**, D360-D370 (2018).
