# Supplementary material for: The microRNA cluster C19MC confers differentiation potential into trophoblast lineages upon human pluripotent stem cells
Source: Nat Commun. 2022 Jun 2;13:3071. doi: 10.1038/s41467-022-30775-w (PMC9163035; doi:10.1038/s41467-022-30775-w)
Supplement: Supplementary file 3 — Description of Additional Supplementary Files [file 41467_2022_30775_MOESM3_ESM.pdf]

## Description of Additional Supplementary Files

**Supplementary Movie 1. Time-lapse imaging of hTS#1 cultured in EVT medium.**

Phase-contrast images were taken from 24 to 120 hours after the cells were seeded. The scale bar indicates 300 μm.

**Supplementary Movie 2. Time-lapse imaging of hTSL$^{naïve}$#1 cultured in EVT medium.**

Phase-contrast images were taken from 24 to 120 hours after the cells were seeded. The scale bar indicates 300 μm.

**Supplementary Movie 3. Time-lapse imaging of hTSL$^{primed}$#1 cultured in EVT medium.**

Phase-contrast images were taken from 24 to 120 hours after the cells were seeded. The scale bar indicates 300 μm.

**Supplementary Data 1. Transcriptome profiling by RNA-seq.**

The following cells were analyzed: primed and naïve hES cells, hTS cells, hTSL$^{primed}$ cells, hTSL$^{naïve}$ cells, EVT-like cells derived from hTS and hTSL$^{naïve}$ cells, ST-like cells derived from hTS, hTSL$^{primed}$, and hTSL$^{naïve}$ cells, and hTS cells with deletion of the C19MC DMR. Expression levels of Refseq genes are shown as TPM.

**Supplementary Data 2. DNA methylation levels of placenta-specific and conventional gDMRs in hES, hTS, and hTSL cells.**

WGBS data were used for the calculation of gDMR methylation levels. Mat: maternal allele, Pat: paternal allele. Two hTSL$^{primed}$ cell lines were analyzed and the average methylation levels are indicated.

**Supplementary Data 3. MiRNA expression profiles.**

The following cells were analyzed: primed and naïve hES cells, hTS cells, hTSL$^{primed}$ cells, hTSL$^{naïve}$ cells, hTS cells with deletion of the C19MC DMR, and hTSL$^{C19MC}$ cells. Expression levels of mature miRNAs are shown as RPM.

**Supplementary Data 4. gRNA, PCR primer, and RNA-FISH probe sequences.**
