# Supplementary material for: The microRNA cluster C19MC confers differentiation potential into trophoblast lineages upon human pluripotent stem cells
Source: Nat Commun. 2022 Jun 2;13:3071. doi: 10.1038/s41467-022-30775-w (PMC9163035; doi:10.1038/s41467-022-30775-w)
Supplement: Supplementary file 11 — Reporting Summary [file 41467_2022_30775_MOESM11_ESM.pdf]

nature portfolio

Corresponding author(s): Takahiro Arima, Hiroaki Okae

Last updated by author(s): Apr 12, 2022

# Reporting Summary

Nature Portfolio wishes to improve the reproducibility of the work that we publish. This form provides structure for consistency and transparency in reporting. For further information on Nature Portfolio policies, see our Editorial Policies and the Editorial Policy Checklist.

## Statistics

For all statistical analyses, confirm that the following items are present in the figure legend, table legend, main text, or Methods section.

| n/a | Confirmed | |
|---|---|---|
| ☐ | ☒ | The exact sample size (*n*) for each experimental group/condition, given as a discrete number and unit of measurement |
| ☐ | ☒ | A statement on whether measurements were taken from distinct samples or whether the same sample was measured repeatedly |
| ☐ | ☒ | The statistical test(s) used AND whether they are one- or two-sided<br>*Only common tests should be described solely by name; describe more complex techniques in the Methods section.* |
| ☐ | ☒ | A description of all covariates tested |
| ☐ | ☒ | A description of any assumptions or corrections, such as tests of normality and adjustment for multiple comparisons |
| ☐ | ☒ | A full description of the statistical parameters including central tendency (e.g. means) or other basic estimates (e.g. regression coefficient) AND variation (e.g. standard deviation) or associated estimates of uncertainty (e.g. confidence intervals) |
| ☐ | ☒ | For null hypothesis testing, the test statistic (e.g. *F*, *t*, *r*) with confidence intervals, effect sizes, degrees of freedom and *P* value noted<br>*Give P values as exact values whenever suitable.* |
| ☒ | ☐ | For Bayesian analysis, information on the choice of priors and Markov chain Monte Carlo settings |
| ☒ | ☐ | For hierarchical and complex designs, identification of the appropriate level for tests and full reporting of outcomes |
| ☒ | ☐ | Estimates of effect sizes (e.g. Cohen's *d*, Pearson's *r*), indicating how they were calculated |

*Our web collection on statistics for biologists contains articles on many of the points above.*

## Software and code

Policy information about availability of computer code

| | |
|---|---|
| Data collection | Illumina's sequencers (HiSeq 2500 and HiSeq X Ten) were used for data collection. |
| Data analysis | TrimGalore (v0.6.5)<br>STAR (v2.7.3a)<br>fastp (v0.20.1)<br>Bowtie 2 (v2.1.0 and v2.3.2)<br>miRBase (v22)<br>featureCounts (v1.6.4)<br>LiftOver tool (https://genome.ucsc.edu/cgi-bin/hgLiftOver)<br>Bismark (v0.19.1)<br>IGV (v2.8.0)<br>R (v3.1.3 and v4.1.2) |

For manuscripts utilizing custom algorithms or software that are central to the research but not yet described in published literature, software must be made available to editors and reviewers. We strongly encourage code deposition in a community repository (e.g. GitHub). See the Nature Portfolio guidelines for submitting code & software for further information.

## Data

Policy information about availability of data

All manuscripts must include a data availability statement. This statement should provide the following information, where applicable:

- Accession codes, unique identifiers, or web links for publicly available datasets
- A description of any restrictions on data availability
- For clinical datasets or third party data, please ensure that the statement adheres to our policy

- The RNA-seq, WGBS, and miRNA-seq data are deposited in DDBJ/GenBank/EMBL under the accession number DRA013428. The ChIP-seq data are deposited in Japanese Genotype-phenotype Archive (JGA) under the accession number JGAS000107. Expression data of Refseq genes and miRNAs are provided with Supplementary Data 1 and 3, respectively. The methylation levels of each CpG site in hTSLprimed cells are deposited in DDBJ Genomic Expression Archive (GEA) under the accession number E-GEAD-474.
- The following publicly available WGBS data were used:  hTS cells (JGAS000107 and JGAS000112), naïve hES cells (GSM2041698 and GSM2041699), hTSLnaïve cells (GSM4525520 and GSM4525521), and primed hES cells (GSM706059 and GSM706060).
- The following databases were used: ConsensusPathDB human pathway database (http://cpdb.molgen.mpg.de/) and mirDIP (http://ophid.utoronto.ca/mirDIP/).
- Raw data of Figs. 2a, 2e, 2f, 3g, 4f, 4g, 4i, 4j, 5c, 5f, 5j, and 5k, and Supplementary Figs. 3c, 4c, and 5i are provided as a Source Data file.

# Field-specific reporting

Please select the one below that is the best fit for your research. If you are not sure, read the appropriate sections before making your selection.

☒ Life sciences ☐ Behavioural & social sciences ☐ Ecological, evolutionary & environmental sciences

For a reference copy of the document with all sections, see nature.com/documents/nr-reporting-summary-flat.pdf

# Life sciences study design

All studies must disclose on these points even when the disclosure is negative.

| | |
|---|---|
| Sample size | No statistical methods were used to predetermine the experimental sample size. Sample sizes were chosen based on previously published sample sizes for similar experiments and analyses in literature (Cinkornpumin et al.,  Stem Cell Reports, 2020; Dong et al., eLife, 2020). Sample sizes for all data sets are clearly indicated in Methods and Figure legends. |
| Data exclusions | No data were excluded from the analyses. |
| Replication | At least two cell lines were used for all experiments, which showed successful replication. The number of independent biological replicates for all experiment are clearly indicated in Methods and Figure legends. |
| Randomization | Sample groups were compared with no randomization. Randomization was not applicable because defined genotype/sample sets were compared. |
| Blinding | No blinding was used in these experiments because the same investigator designed and conducted the experiments, including the appropriate controls. |

# Reporting for specific materials, systems and methods

We require information from authors about some types of materials, experimental systems and methods used in many studies. Here, indicate whether each material, system or method listed is relevant to your study. If you are not sure if a list item applies to your research, read the appropriate section before selecting a response.

### Materials & experimental systems

| n/a | Involved in the study | |
|---|---|---|
| ☐ | ☒ | Antibodies |
| ☐ | ☒ | Eukaryotic cell lines |
| ☒ | ☐ | Palaeontology and archaeology |
| ☒ | ☐ | Animals and other organisms |
| ☒ | ☐ | Human research participants |
| ☒ | ☐ | Clinical data |
| ☒ | ☐ | Dual use research of concern |

### Methods

| n/a | Involved in the study | |
|---|---|---|
| ☐ | ☒ | ChIP-seq |
| ☒ | ☐ | Flow cytometry |
| ☒ | ☐ | MRI-based neuroimaging |

## Antibodies

| | |
|---|---|
| Antibodies used | All antibodies used in the study were obtained from manufacturers. |

| | |
|---|---|
| Antibodies used | The manufacturers, catalog numbers, dilution rates, and usages of primary antibodies are listed below.<br>Anti-TP63 (Cell Signaling, #13109; 1:100 for Immunostaining)<br>Anti-TFAP2C (Santa Cruz Biotechnology, #sc-12762; 1:200 for Immunostaining)<br>Ant-KRT7 (Abcam, #ab119697; 1:100 for Immunostaining)<br>Anti-hCG (DAKO, #IS508; 1:10 for Immunostaining)<br>Anti-KLF17 (Sigma, #HPA024629; 1:200 for Immunostaining)<br>Anti-H3K4me3 (MBL, #MABI0304; 0.8ng/ul for ChIP)<br>Anti-ELF5 (Sigma, #HPA062706; 1:100 for Immunostaining)<br>Anti-TEAD4 (Abcam, #ab58310; 1:100 for Immunostaining)<br>Anti-CDX2 (Abcam, #ab157524; 1:100 for Immunostaining)<br>PE-conjugated anti-OCT-4A (Cell Signaling, #56159; 1:800 for Immunostaining)<br>PE-conjugated anti-HLA-G (Abcam, #ab24384; 1:50 for Flow cytometry, 1:200 for Immunostaining)<br>PE-conjugated anti-SDC1 (Miltenyi Biotec, #130-119-928; 1:500 for Immunostaining)<br>Alexa Fluor 488-conjugated anti-HLA-ABC (Biolegend, #311415; 1:50 for Flow cytometry) |
| Validation | All antibodies were validated by their manufacturers for the applications and species used in this study. Validation statements and literature citations are available on the manufacturer's websites.<br>Anti-TP63 (Cell Signaling, #13109): https://en.cellsignal.jp/products/primary-antibodies/p63-a-d2k8x-xp-rabbit-mab/13109<br>Anti-TFAP2C (Santa Cruz Biotechnology, #sc-12762): https://www.scbt.com/ja/p/ap-2gamma-antibody-6e4-4<br>Ant-KRT7 (Abcam, #ab119697): https://www.abcam.com/cytokeratin-7-antibody-sp52-ab119697.html<br>Anti-hCG (DAKO, #IR508): https://www.agilent.com/cs/library/packageinsert/public/SSIR508CEEFG_01.pdf<br>Anti-KLF17 (Sigma, #HPA024629): https://www.sigmaaldrich.com/JP/en/product/sigma/hpa024629<br>Anti-H3K4me3 (MBL, #MABI0304): https://labchem-wako.fujifilm.com/us/product/detail/W01M1630-3481.html<br>Anti-ELF5 (Sigma, #HPA062706): https://www.sigmaaldrich.com/JP/en/product/sigma/hpa062706<br>Anti-TEAD4 (Abcam, #ab58310): https://www.abcam.com/tead4-antibody-5h3-ab58310.html<br>Anti-CDX2 (Abcam, #ab157524): https://www.abcam.com/cdx2-antibody-cdx2-88-ab157524.html<br>PE-conjugated anti-OCT-4A (Cell Signaling, #56159): https://en.cellsignal.jp/products/antibody-conjugates/oct-4a-c30a3-rabbit-mab-pe-conjugate/56159<br>PE-conjugated anti-HLA-G (Abcam, #ab24384): https://www.abcam.com/pe-hla-g-antibody-mem-g9-ab24384.html<br>PE-conjugated anti-SDC1 (Miltenyi Biotec, #130-119-928): https://www.miltenyibiotec.com/JP-en/products/cd138-antibody-anti-human-44f9.html<br>Alexa Fluor 488-conjugated anti-HLA-ABC (Biolegend, #311415): https://www.biolegend.com/en-us/search-results/alexa-fluor-488-anti-human-hla-a-b-c-antibody-2899 |

# Eukaryotic cell lines

Policy information about cell lines

| | |
|---|---|
| Cell line source(s) | hES cell lines (SEES1 and SEES4) were generated from Akutsu et al., Regen. Ther., 2015. hTS cell lines (CT27, CT29, and CT30) were established in our previous study (Okae et al., Cell Stem Cell, 2018). |
| Authentication | hES cell lines (SEES1 and SEES4) were derived and authenticated by Drs. Hidenori Akutsu and Akihiro Umezawa (The National Center for Child Health and Development, Tokyo, Japan). hTS cell lines (CT27, CT29, and CT30) were derived and authenticated by Takahiro Arima and Hiroaki Okae (Tohoku University Graduate School of Medicine, Sendai, Japan). |
| Mycoplasma contamination | All cell lines were tested negative for mycoplasma contamination. |
| Commonly misidentified lines<br>(See ICLAC register) | No commonly misidentified cell line was used in this study. |

# ChIP-seq

## Data deposition

☒ Confirm that both raw and final processed data have been deposited in a public database such as GEO.

☒ Confirm that you have deposited or provided access to graph files (e.g. BED files) for the called peaks.

| | |
|---|---|
| Data access links<br>*May remain private before publication.* | The ChIP-seq data are deposited at JGA under the accession number JGAS000107. |
| Files in database submission | CT27_H3K4me3_R1.fastq.gz, CT27_H3K4me3_R2.fastq.gz, CT27_Input_R1.fastq.gz, CT27_Input_R2.fastq.gz |
| Genome browser session<br>(e.g. UCSC) | n/a |

## Methodology

| | |
|---|---|
| Replicates | ChIP-seq was performed on two independent hTS cell lines, CT27 and TSblast-1. The data were highly correlated between these two cell lines, and only the data of CT27 is shown in the manuscript. |
| Sequencing depth | Total number of reads: 23,372,186<br>Uniquely mapped reads: 22,912,465<br>Length of reads: 101 (paired) |

| | |
|---|---|
| Antibodies | H3K4me3: MBL clone #MABI0304 |
| Peak calling parameters | macs2 callpeak -t chip.bam -c input.bam -f BAMPE -n sample_name -g hs --to-large --nomodel |
| Data quality | 42,113 peaks were called (q < 0.05) |
| Software | Bowtie2 (v2.2.9)<br>IGV (v2.8.0)<br>MACS2 (v2.1.1) |
